# Control of RNA degradation in cell fate decision

**DOI:** 10.3389/fcell.2023.1164546

**Published:** 2023-03-21

**Authors:** Mingqiang Deng, Xiwei Wang, Zhi Xiong, Peng Tang

**Affiliations:** ^1^ Center for Cell Lineage and Development, Guangzhou Institutes of Biomedicine and Health, Chinese Academy of Sciences, Guangzhou, China; ^2^ Key Laboratory of Biological Targeting Diagnosis, Therapy and Rehabilitation of Guangdong Higher Education Institutes, The Fifth Affiliated Hospital of Guangzhou Medical University, Guangzhou, China; ^3^ Guangzhou Laboratory, Guangzhou, Guangdong, China; ^4^ Bioland Laboratory (Guangzhou Regenerative Medicine and Health GuangDong Laboratory), Guangzhou, China

**Keywords:** RNA degradation, cell fate decision, stem cell, RNA alternative processing, post-transcriptional regulation

## Abstract

Cell fate is shaped by a unique gene expression program, which reflects the concerted action of multilayered precise regulation. Substantial research attention has been paid to the contribution of RNA biogenesis to cell fate decisions. However, increasing evidence shows that RNA degradation, well known for its function in RNA processing and the surveillance of aberrant transcripts, is broadly engaged in cell fate decisions, such as maternal-to-zygotic transition (MZT), stem cell differentiation, or somatic cell reprogramming. In this review, we first look at the diverse RNA degradation pathways in the cytoplasm and nucleus. Then, we summarize how selective transcript clearance is regulated and integrated into the gene expression regulation network for the establishment, maintenance, and exit from a special cellular state.

## Introduction

Beginning with a fertilized egg, numerous identical or distinct cells are continually generated to ensure the proper organization and function of each tissue and organ during the lifespan of multicellular organisms (Tam and Behringer, 1997; Kojima et al., 2014). Deciphering the molecular mechanisms underlying the cell-type specific gene expression program, which ultimately shapes the cell fate and identity, is critically important not only in theory but also in the clinic. Improved knowledge of the regulatory mechanisms in cell fate decisions will deepen our understanding of normal or defective development and provide more detailed guidelines for regenerative medicine.

Pioneer works demonstrated that lineage conversion could be directed simply through the introduction of a specific transcription factor (Davis et al., 1987; Kulessa et al., 1995). In particular, somatic cells can be reprogramed into a pluripotent state *via* ectopic expression of a defined cocktail of transcription factors: Oct4, Klf4, Sox2, and Myc (Takahashi and Yamanaka, 2006). These and other additional lines of evidence convince us that transcription plays an instructive role in cell fate decisions. Consequently, regulation at the level of mRNA synthesis, in the context of transcription factors, epigenetics regulators, non-coding RNAs, and three-dimensional (3D) genome, is the primary research concern in cell fate decisions (Ng and Surani, 2011; Yadav et al., 2018; Stadhouders et al., 2019).

During the process of maternal-to-zygotic transition (MZT), the initial step of early embryo development in which fertilized egg is reprogrammed into a totipotent embryo, a subset of maternal RNAs must be cleared timely and efficiently apart from transcriptionally awakening the zygotic genome. Impediment in maternal RNA clearance by genetic inactivation of the RNA degradation-associated genes *Btg4*, *Pabpn1l*, or *Cnot6l* leads to MZT failure and female infertility in mice (Liu et al., 2016; Yu et al., 2016; Sha et al., 2018; Zhao et al., 2020). A pairwise comparison of the RNA half-lives uncovered a significant discrepancy in the RNA decay rate for the genes uniformly expressed in both induced pluripotent stem (iPS) cells and the differentiated counterpart (Neff et al., 2012), implying a potential role of RNA degradation in pluripotency maintenance or somatic reprogramming. It has been reported that non-sense-mediated RNA decay or RNA N6-methyladenosine (m^6^A) methylation could promote the degradation of specific pluripotency transcripts and facilitate mouse embryonic stem cell differentiation (Batista et al., 2014; Aguilo et al., 2015; Geula et al., 2015; Li et al., 2015), whereas RNA exosome complex restrains human embryonic stem cell differentiation by degrading differentiation-associated transcripts (Belair et al., 2019). In general, these compelling proofs indicate that RNA turnover should be an essential driving force in cell fate decisions.

Here, we briefly introduce the RNA degradation factors and describe how they orchestrate the highly regulated RNA degradation pathways. Furthermore, we outline how selective RNA turnover is regulated and becomes an integral part of the gene regulatory network in cell fate decisions.

### RNA degradation machinery

For the majority of RNA polymerase II (Pol II) transcribed RNAs, a unique 7-methylguanosine (m^7^G) cap will be installed at the 5′ end of nascent RNA (20–25 nucleotides in length), whereas a stretch of non-templated adenosines will be added to the 3′ end (poly(A) tail) (Shatkin and Manley, 2000; Rambout and Maquat, 2020; Passmore and Coller, 2022). m^7^G cap and poly(A) tail act as versatile platforms to recruit diverse effector proteins and hence affect almost all aspects of RNA metabolism, such as RNA decay and translation efficiency (Furuichi et al., 1977; Shimotohno et al., 1977; Drummond et al., 1985; Bernstein et al., 1989; Caponigro and Parker, 1995; Rambout and Maquat, 2020; Passmore and Coller, 2022). Transcripts with unprotected ends will be swiftly removed by the RNA exonucleases. RNA exonucleases, together with many other core factors and co-factors of the RNA degradation machinery, orchestrate the RNA degradation pathways (Supplementary Table S1).

### RNA exonucleases

During the whole life cycle, RNA is subject to surveillance by the RNA degradation machinery. RNA exonucleases clear the RNAs with exposed free ends in the 5′-to-3′ or 3′-to-5′ direction. In mammals, XRN1 and XRN2, located in the cytoplasm and nucleus, respectively, catalyze the 5′-to-3′ RNA hydrolysis processively (Nagarajan et al., 2013). Despite the difference in cellular localization, structure analysis reveals that XRN1 and XRN2 share an extensively conserved N-terminal domain, which is responsible for the exonucleolytic digestion of RNA with 5′-monophosphorylated ends, once activated by divalent cations (Jinek et al., 2011; Nagarajan et al., 2013; Overbeck et al., 2022).

Conversely, 3′-to-5′ RNA degradation is catalyzed by the multi-subunit complex, RNA exosome (Puno et al., 2019). The structure and composition of the RNA exosome are well conserved across species, wherein nine proteins form the barrel-shaped catalytically inactive core (EXO9), whereas the two catalytic subunits EXOSC10 and DIS3 (or DIS3L) are placed on the top and bottom of the EXO9 core, respectively (Zinder et al., 2016; Weick et al., 2018; Puno et al., 2019). EXOSC10 is a distributive 3′-to-5′ exonuclease, predominantly located in the nucleus, and trims RNAs with the 3′-hydroxyl terminus. By contrast, DIS3/DIS3L is a processive 3′-to-5′ exonuclease and digests RNAs with either 3′-hydroxyl or 3′-phosphate terminus. The majority of DIS3 resides in the nucleus, whereas DIS3L exclusively locates in the cytoplasm (Januszyk and Lima, 2014; Zinder et al., 2016; Puno et al., 2019). Acute protein depletion assays revealed that DIS3 principally contributes to the degradation of enhancer RNAs (eRNAs), promoter upstream transcripts (PROMPTs), and products of premature cleavage and polyadenylation (PCPA) in the nucleoplasm, whereas EXOSC10 primarily facilitates the trimming of short 3′ extended ribosomal and small nucleolar RNAs located in the nucleolus (Davidson et al., 2019).

### Decapping and deadenylation complexes

As mentioned previously, RNA exonucleases can only attack RNA with exposed free 5′ or 3′ terminus. Thus, the cleavage of the 5′ cap structure (decapping) and/or removal of the 3′ poly(A) tail (deadenylation) is usually a prerequisite for RNA degradation.

Two factors, DCP1 and DCP2, act together as the decapping holoenzyme to cleave the m^7^G cap and then release 5′ m^7^GDP and 3′ fragment with monophosphate at the 5′ terminus. DCP2 specifically recognizes m^7^G-cap or m^2,2,7^G-cap and catalyzes their cleavage through its NUDIX domain, a motif shared in pyrophosphatases. However, DCP1 functions as a coactivator to enhance the decapping activity of DCP2 and bridges the interaction of the decapping complex with other co-factors (Ling et al., 2011; Vidya and Duchaine, 2022).

Compared with RNA decapping, RNA deadenylation seems more complicated that involves the PAN2–PAN3 and CCR4–NOT complexes, both functioning specifically on poly(A) sequences. A biphasic model is proposed for deadenylation: in the initial phase, poly(A)-binding protein PABPC1 facilitates the loading of the PAN2–PAN3 complex to remove the distal part of poly(A) tail through the distributive exonuclease PAN2. In the second, fast phase, the CCR4–NOT complex relays and digests the residual adenosines to a very few adenosines *via* the catalytic subunits CCR4 and CAF1 (Passmore and Coller, 2022).

### RNA endonucleases

Although RNA exonucleases are required for complete RNA destruction, decapping and deadenylation are not essential. RNA exonucleolytic decay could be initiated at internal endonucleolytic sites within the RNA body (Coller and Parker, 2004; Tomecki and Dziembowski, 2010; Schoenberg, 2011). The RNA exonuclease DIS3, instead of its cytoplasmic paralog DIS3L, displays endonucleolytic activity as well, which may cooperate with its exonuclease domain to clear RNAs more efficiently (Schaeffer et al., 2009; Schneider et al., 2009; Staals et al., 2010; Tomecki and Dziembowski, 2010; Tomecki et al., 2010). Cumulative evidence shows that RNA endonucleases have emerged as crucial modulators of gene expression (Tomecki and Dziembowski, 2010; Schoenberg, 2011).

Non-sense-mediated mRNA decay (NMD) and microRNAs (miRNAs)/small interfering RNA (siRNA)-mediated RNA interference (RNAi) in the cytoplasm may be the best-characterized cases involving the endonuclease activity. NMD is a quality control mechanism employed to eliminate transcripts harboring a premature termination codon (PTC) (Tomecki and Dziembowski, 2010; Schoenberg, 2011; Kurosaki et al., 2019). If a PTC locates ≥50–55 nucleotides (nt) upstream of an exon junction complex (EJC), the complex assembling ∼24 nt upstream of the exon–exon junction following splicing, will induce ribosome stalling and sequentially activate the cascade of NMD to recruit the SMG5/SMG7 heterodimer or SMG6. SMG5 and SMG7 can recruit RNA decapping and deadenylation complex, whereas SMG6 can trigger endonucleolytic cleavage at the sites around the PTC (Kurosaki et al., 2019). These activities will be unified to initiate the clearance of faulty RNA substrates (Huntzinger et al., 2008; Eberle et al., 2009; Colombo et al., 2017; Boehm et al., 2021). Additionally, it was reported that transcripts with a 5′ upstream open reading frame (uORF) or an unusually long 3′ untranslated region (3′ UTR) could be NMD targets (Han et al., 2018; Kurosaki et al., 2019).

During RNAi, miRNA/siRNA associates with Argonaute (Ago) family protein and GW182 to form the functional RNA-induced silencing complex (RISC). Then, it silences its target expression by repressing translation or accelerating mRNA degradation, the latter of which is proved to be the major function of miRNAs in mammalian cells (Guo et al., 2010; Jonas and Izaurralde, 2015). Mechanistically, RISC could facilitate RNA deadenylation through the interaction of GW182 with the subunits of the deadenylation complexes, namely, PAN3, NOT1, and NOT9 (Jonas and Izaurralde, 2015). Alternatively, in the case of perfect base-pairing between miRNA/siRNA and its target, the endonucleolytic cleavage is favored *via* the slicing activity of the Ago2 protein (Valencia-Sanchez et al., 2006).

On the contrary, nuclear RNA endonucleases are under-reported. The Integrator complex is a metazoan-specific complex, originally identified in the biogenesis of non-coding RNA, such as small nuclear RNAs (snRNAs) and eRNAs (Lai et al., 2015). Recently, it was demonstrated that the Integrator would trigger premature transcription termination of many protein-coding genes through the endonucleolytic cleavage of the nascent transcripts (Elrod et al., 2019; Tatomer et al., 2019; Lykke-Andersen et al., 2021; Stein et al., 2022). In another scenario, Polycomb repressive complexes (PRCs) could recruit the Rixosome complex to the promoters of PRC target genes to silence their expression *via* endonucleolytic cleavage of the nascent RNAs, analogous to the Integrator complex (Zhou et al., 2022).

### RNA degradation pathway

**FIGURE 1:**
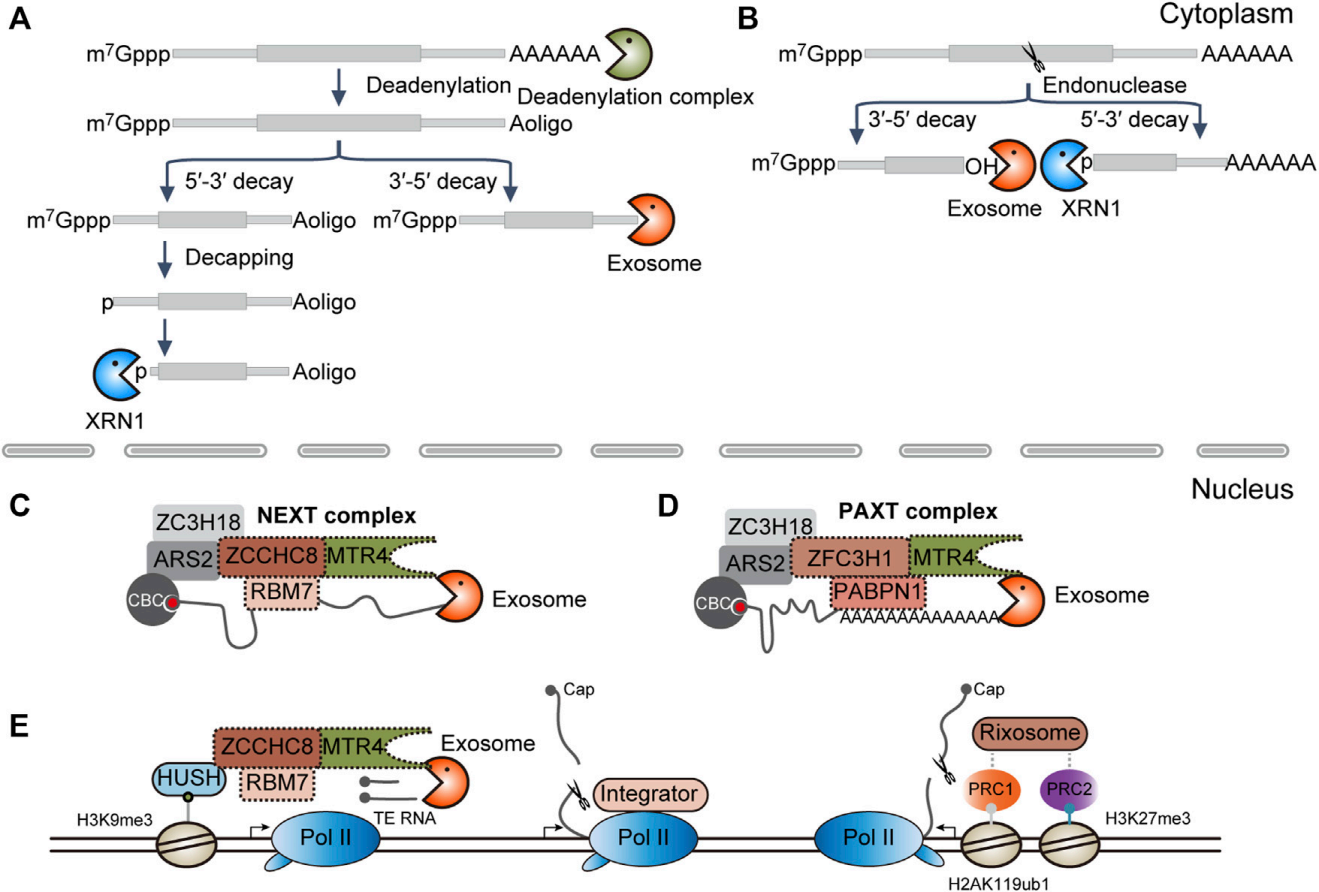
Diverse RNA decay pathways. **(A)** RNA exonucleolytic decay pathways in the cytoplasm. XRN1, 5′-to-3′ RNA exonuclease; exosome, 3′-to-5′ RNA exonuclease complex. **(B)** RNA endonucleolytic decay pathways in the cytoplasm. **(C)** NEXT complex-mediated RNA decay in the nucleus. CBC, cap-binding complex, composed of CBC20 and CBC80; NEXT, nuclear exosome targeting complex, composed of ZCCHC8, RBM7, and MTR4. **(D)** PAXT complex-mediated RNA decay in the nucleus. PAXT, PolyA tail exosome targeting complex, composed of ZFC3H1, PABPN1, and MTR4. **(E)** Co-transcriptional RNA decay pathways. Human silencing hub (HUSH) complex functions in the maintenance of the H3K9me3 modification. HUSH is composed of the chromodomain containing proteins MPP8, TASOR, and PPHLN1, in which MPP8 can interact directly with ZCCHC8, one subunit of the NEXT complex; Pol II, RNA polymerase II; Integrator is composed of 14 subunits, in which IntS11 along with IntS4 and 9 make up the endonucleolytic cleavage module. Integrator can directly bind to Pol II; PRC, Polycomb repressive complex; here, PRC1 and PRC2 can catalyze the mono-ubiquitination of histone H2A lysine 119 (H2AK119ub1) and tri-methylation of H3 lysine 27 (H3K27me3), respectively. Rixosome is composed of seven subunits, in which LAS1L is the endonucleolytic subunit, whereas the TEX10 subunit can interact with the PRC complex.

Most of our knowledge about RNA degradation pathways is from mRNA decay in the cytoplasm. In mammals, cytoplasmic mRNA degradation is usually initiated by deadenylation. Then, RNAs will be directly cleared through the 3′-to-5′ RNA decay pathway or will be decapped and followed by 5′-to-3′ degradation (Figure 1A). In terms of RNA endonucleases, such as SMG6 and Ago2, the transcripts are cleaved into the 5′ and 3′ fragments. The resulting intermediates will be subject to further clearance by cytoplasmic RNA exosome and XRN1, respectively (Figure 1B) (Coller and Parker, 2004).

By contrast, RNA degradation in the nucleus does not receive much attention. It has been demonstrated that the decapping complex is implicated in the degradation of U3 and U8 snoRNA in the nucleolus (Gaviraghi et al., 2018), Tsix RNA in the chromatin (Aeby et al., 2020), and nascent RNAs near the promoter-proximal pause sites (Brannan et al., 2012). However, decapping-dependent 5′-to-3′ RNA decay may not be a general nuclear RNA degradation way as RNA decapping usually requires the synergistic action of many auxiliary factors, most of which are concentrated in the cytoplasmic P body (Supplementary Table S1) (Coller and Parker, 2004; Ling et al., 2011; Vidya and Duchaine, 2022). Instead, the nuclear cap-binding complex (CBC) CBC20-CBC80 initially recognizes the 5′ m^7^G cap, which in turn recruits the polyA tail exosome targeting (PAXT) or nuclear exosome targeting (NEXT) complex through the CBC-ARS2-ZC3H18 axis and serves to target the RNA substrates for degradation by nuclear RNA exosome (Figures 1C, D) (Garland and Jensen, 2020; Ogami and Suzuki, 2021). Alternatively, analogous to the described Integrator or Rixosome complex, RNA decay machinery may be co-transcriptionally recruited to the chromatin by the transcription machinery or histone modifiers for the degradation of target genes (Figure 1E) (Elrod et al., 2019; Tatomer et al., 2019; Lykke-Andersen et al., 2021; Garland et al., 2022; Stein et al., 2022; Zhou et al., 2022).

### RNA degradation regulation in cell fate decision

As the general RNA decay machineries exhibit little substrate specificity, selective RNA degradation is determined largely by specific sequence features encoded in the RNA sequence and the cognate RNA-binding proteins (RBPs). Additionally, it is evidenced that RNA degradation is tightly interconnected with RNA processing (Tian and Manley, 2017; Kurosaki et al., 2019). In the following paragraphs, we will discuss how RNA degradation is regulated to specify cell fate.

### Interplay between RNAs and RBPs dictates RNA decay in cell fate decision

#### RBP-mediated RNA decay regulation in cell fate decision

Approximately 1,900 and 1,400 proteins are cataloged as RBPs in *Homo sapiens* and *Mus musculus*, respectively, or more in other new datasets (Hentze et al., 2018). In principle, RBPs could bind to a specific sequence and/or structural motif in the RNA *via* their canonical or non-conventional RNA-binding domains (Hentze et al., 2018) and sequentially recruit different commitment proteins or complex, such as RNA degradation machineries to regulate the fate of bound RNAs (He et al., 2022).

**FIGURE 2:**
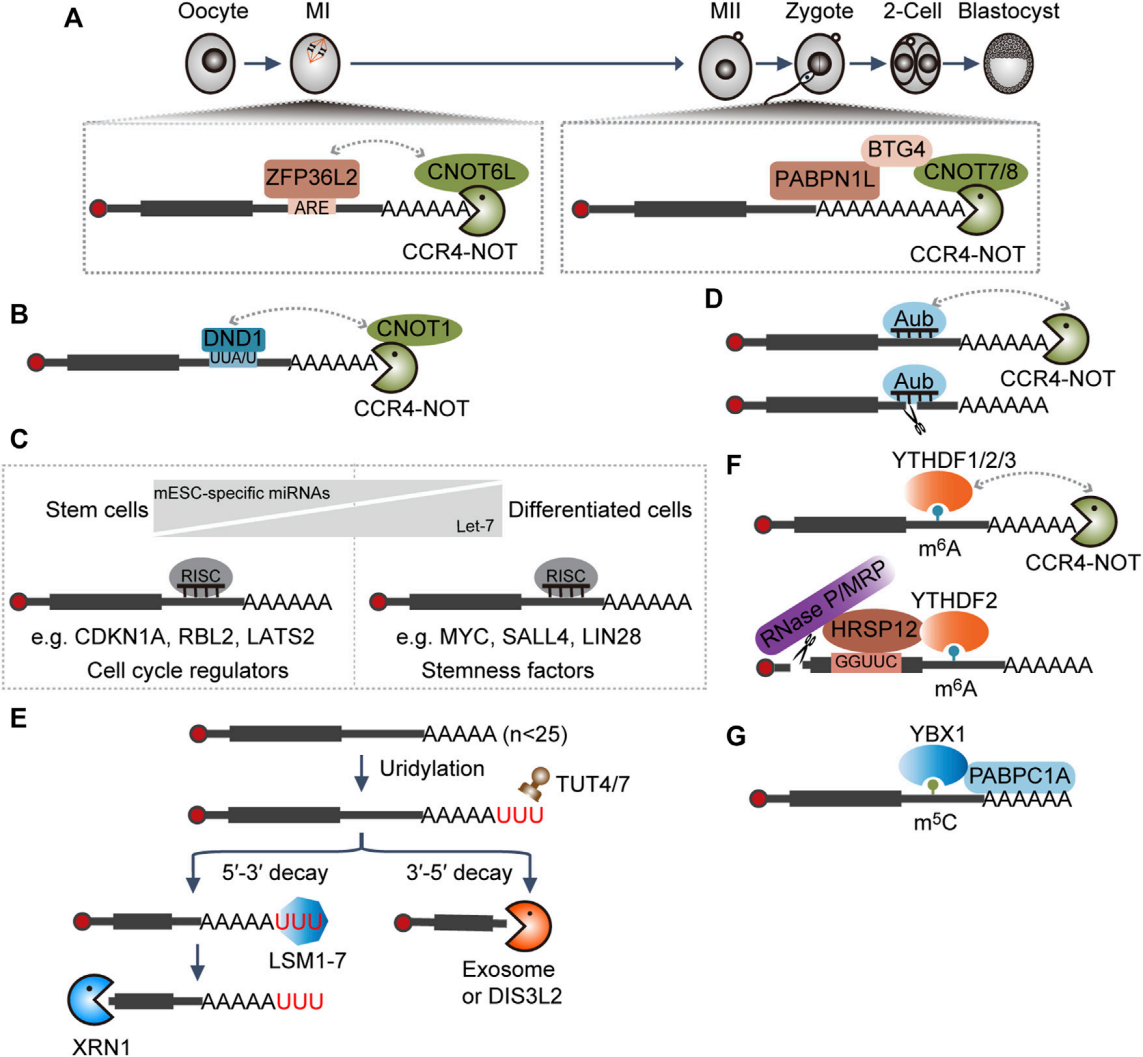
Interplay between RNAs and RNA-binding proteins (RBPs) dictates RNA decay. **(A,B)** RBP-mediated RNA degradation. ARE, AU-rich element; CCR4–NOT, RNA deadenylation complex. **(C)** miRNA-mediated RNA degradation. miRNA, microRNA; RISC, RNA-induced silencing complex; mESC-specific miRNAs, miRNAs specifically expressed in mouse embryonic stem cells: miR-291a-3p, miR-291b-3p, miR-294, miR-295, and miR-302. **(D)** piRNA-mediated RNA degradation. piRNA, Piwi-interacting RNA; Aubergine (Aub), the cytoplasmic Piwi proteins in *Drosophila.* **(E)** RNA uridylation-mediated RNA degradation. TUT4/7, RNA terminal uridylyltransferases; LSM1-7, a complex that especially recognizes the substrate with 3′ terminal oligoA tail and functions in facilitating RNA decapping; DIS3L2, RNA 3′-to-5′ exonuclease that especially functions in clearing RNAs with 3′ U- tail; XRN1, 5′-to-3′ RNA exonuclease; exosome, 3′-to-5′ RNA exonuclease complex. **(F)** m^6^A-modification-mediated RNA degradation. m^6^A, N6-methyladenosine; RNase P/MRP, a complex with endonucleolytic activity. **(G)** m^5^C-modification-mediated RNA stabilization. m^5^C, 5-methylcytosine.

Upon oocyte meiotic resumption during mouse oocyte maturation, the MAPK signal pathway will be activated to extend the length of poly(A) tails of many maternal transcripts. Then, these transcripts will be activated translationally to produce more proteins, among which various RNA degradation factors are reported such as ZFP36L2, CNOT6L, CNOT7, BTG4, and PABPN1L (Liu et al., 2016; Yu et al., 2016; Sha et al., 2018; Zhao et al., 2020). At the early stage, increased ZFP36L2 recognizes AU-rich elements (AREs) containing transcripts and functions as an adapter to recruit the CCR4–NOT complex through interaction with the CNOT6L subunit (Sha et al., 2018). Later, PABPN1L specifically binds to the poly(A) tails and interacts with BTG4 to recruit the CCR4–NOT complex *via* the association with the CNOT7/8 subunit (Figure 2A) (Yu et al., 2016; Zhao et al., 2020). Together, they contribute to maternal transcripts clearance during oocyte maturation and MZT by accelerating deadenylation. Embryos with either BTG4 or PABPN1L depletion will be arrested at the 1∼2-cell stage and characterized by female infertility (Liu et al., 2016; Yu et al., 2016; Zhao et al., 2020).

Murine primordial germ cells (PGCs) are first identified at the base of the incipient allantois around embryonic day (E) 7.25 (Saitou and Yamaji, 2012). *Dnd1* is transcriptionally activated during the stage E6.5–E6.75 in PGC precursors (Yabuta et al., 2006). DND1 could directly bind to transcripts with a UU(A/U) trinucleotide motif at the 3′ UTRs and then target substrates for degradation through recruiting the CCR4–NOT complex (Figure 2B). Especially, DND1 could preferentially suppress the expression of the regulators associated with apoptosis and inflammation, which is critical for the maintenance of the self-renewal of PGCs (Yamaji et al., 2017).

#### Small RNA-mediated RNA decay regulation in cell fate decision

Small RNAs of 20–30 nucleotides can be classified into three major classes—miRNAs, endogenous siRNAs (endo–siRNAs), and Piwi-interacting RNAs (piRNAs)—which differ in their biogenesis pathways and associated Argonaute-family proteins (Ghildiyal and Zamore, 2009; Kim et al., 2009). Generally, miRNAs/siRNAs could assemble with the Ago-subfamily proteins into the RISC complex and then target the base-paired targets for translation repression or RNA degradation, whereas piRNAs interact with Piwi-subfamily proteins and commonly function in transposon silencing (Ghildiyal and Zamore, 2009; Kim et al., 2009; Wang et al., 2022).

miRNAs can be further divided into canonical and non-canonical miRNAs; the former are initially transcribed as primary miRNAs and then processed into hairpin-shaped precursor (pre-miRNA) by the microprocessor, composed of DROSHA and DGCR8, in the nucleus. Subsequently, the resulting pre-miRNAs are exported into the cytoplasm, where they will be further cleaved by DICER into the miRNA duplex and assembled into the RISC complex (Ghildiyal and Zamore, 2009; Kim et al., 2009). Mouse embryonic stem cells (mESCs) with *Dgcr8* or *Dicer* knockout (KO) showed defects in proliferation and differentiation (Kanellopoulou et al., 2005; Murchison et al., 2005; Wang et al., 2007), indicating a dual role of miRNAs in pluripotency maintenance and differentiation. Mechanistically, mESC-specific miRNAs (miR-291a-3p, miR-291b-3p, miR-294, miR-295, and miR-302) shared similar seed regions and acted redundantly to reduce the level of *Cdkn1a*, *Rbl2*, and *Lats2*, negative regulators of G1-S cell cycle transition, thereby sustaining the high proliferation rate of mESCs (Wang Y. et al., 2008). Upon differentiation, mESC-specific miRNAs are downregulated, whereas mature let-7 are upregulated. Then, let-7 will bind to and facilitate the degradation of pluripotency-associated genes *Myc*, *Sall4*, and *Lin28*, thereby promoting mESC differentiation (Figure 2C) (Melton et al., 2010).

piRNAs are 23–31 nucleotides in length and are generated from single-stranded transcripts independent of DICER (Vagin et al., 2006; Wang et al., 2022). In addition to the prominent role in transposon silencing, increasing evidence shows that the Piwi–piRNA complex is also implicated in the regulation of the stability or translation efficiency of protein-coding genes in germ cells (Wang et al., 2022). In early *Drosophila* embryos, piRNAs in complex with cytoplasmic Piwi protein Aubergine (Aub) or Argonaute 3 (Ago3) could target and direct the degradation of many maternal mRNAs involved in germ cell development by either direct endonucleolytic cleavage or recruitment of the CCR4–NOT deadenylation complex (Figure 2D) (Rouget et al., 2010; Barckmann et al., 2015).

endo-siRNAs are generated directly from long double-stranded RNAs by Dicer (Ghildiyal and Zamore, 2009; Kim et al., 2009). They co-exist with miRNAs and piRNAs in mouse oocytes (Tam et al., 2008; Watanabe et al., 2008). Mouse oocytes with Dicer but not Dgcr8 depletion showed meiotic arrest, accompanied by the dysregulation of many transcripts (Murchison et al., 2007; Tang et al., 2007; Suh et al., 2010), underscoring the essential role of endo-siRNAs in oogenesis. However, the exact transcripts for degradation remain unclear.

#### RNA modification-mediated RNA decay regulation in cell fate decision

Apart from the canonical 5′ m^7^G cap and 3′ poly(A) tail modifications, RNAs are extensively decorated at the 3′ terminus or internal sites by other RNA modification enzymes, which have multifaceted roles in RNA metabolism, including RNA decay (Li and Mason, 2014; Yu and Kim, 2020).

TUT4 and TUT7 are terminal uridylyltransferases, which function redundantly in uridylating mRNAs with short poly(A) tails (shorter than ∼25 nucleotides) (Lim et al., 2014). The LSM1–7 complex binds to short poly(A) tails with terminal uridylyl residues more efficiently and facilitates the assembly of the decapping complex (Chowdhury et al., 2007; Song and Kiledjian, 2007). Decapped mRNAs are then subject to degradation by XRN1, or alternatively, RNA exosome or DIS3L2 will digest the RNA from the 3′ end (Figure 2E) (Lim et al., 2014). Mice with *Tut4–Tut7* double-knockout failed to eliminate some maternal transcripts during oocyte maturation and cannot generate functional MII oocytes, thereby resulting in female infertility (Morgan et al., 2017; Chang et al., 2018).

m^6^A, the most abundant internal modification in mRNAs, can be recognized by diverse readers to mediate different biological activities (Li and Mason, 2014). In the cytoplasm, YTHDF1/2/3 proteins redundantly bind to the same m^6^A-modified mRNAs and directly recruit the CCR4–NOT deadenylase complex (Du et al., 2016; Zaccara and Jaffrey, 2020). In some instances, HRSP12 can bind to specific sequences upstream of the m^6^A sites and facilitate the association between YTHDF2 and RNA endonuclease complex RNase P/MRP (Park et al., 2019). These activities, alone or together, contribute to accelerated RNA decay (Figure 2F) (Wang et al., 2014; Du et al., 2016; Park et al., 2019; Zaccara and Jaffrey, 2020). It was found that pluripotency factors, such as *Nanog*, *Sox2*, *Klf4*, and *c-Myc*, are modified by m^6^A, ensuring their timely clearance and hence efficient exit from the self-renewal state during differentiation (Batista et al., 2014; Aguilo et al., 2015; Geula et al., 2015).

During MZT of zebrafish embryogenesis, Y-box-binding protein 1 (Ybx1) preferentially binds to a subset of maternal mRNAs with 5-methylcytosine (m^5^C) modifications and protects them from degradation through the recruitment of the poly(A) tail-binding protein Pabpc1a (Figure 2G) (Yang et al., 2019), which ensures the production of sufficient associated proteins to support normal embryogenesis (Yang et al., 2019).

### RNA alternative processing coupled RNA decay in cell fate decision

#### Alternative splicing coupled NMD in cell fate decision

As much as 95% of multiexon genes undergo alternative splicing in humans (Wang E. T. et al., 2008; Pan et al., 2008), greatly expanding the human proteome. Furthermore, transcriptome analysis revealed that ∼30%–35% of the splicing events in human and mouse cells will introduce PTCs and thus can be NMD targets (Lewis et al., 2003; Pan et al., 2006; Weischenfeldt et al., 2012). Alternative splicing is dynamically regulated and coupled with NMD to regulate gene expression in diverse physiological activities (Kurosaki et al., 2019).

Mice with NMD factors KO typically suffered from embryonic lethality within the stage E5.5–E9.5, during which the three different germ layers’ specialization initiates, implying that NMD may function in the progress from pluripotency toward differentiation (Medghalchi et al., 2001; Weischenfeldt et al., 2008; McIlwain et al., 2010; Li et al., 2015; Han et al., 2018). In line with this, it was expounded that the exit from naïve pluripotency was delayed if NMD-associated factors were depleted in the mESCs (Leeb et al., 2014; Li et al., 2015; Lackner et al., 2021; Huth et al., 2022).

**FIGURE 3:**
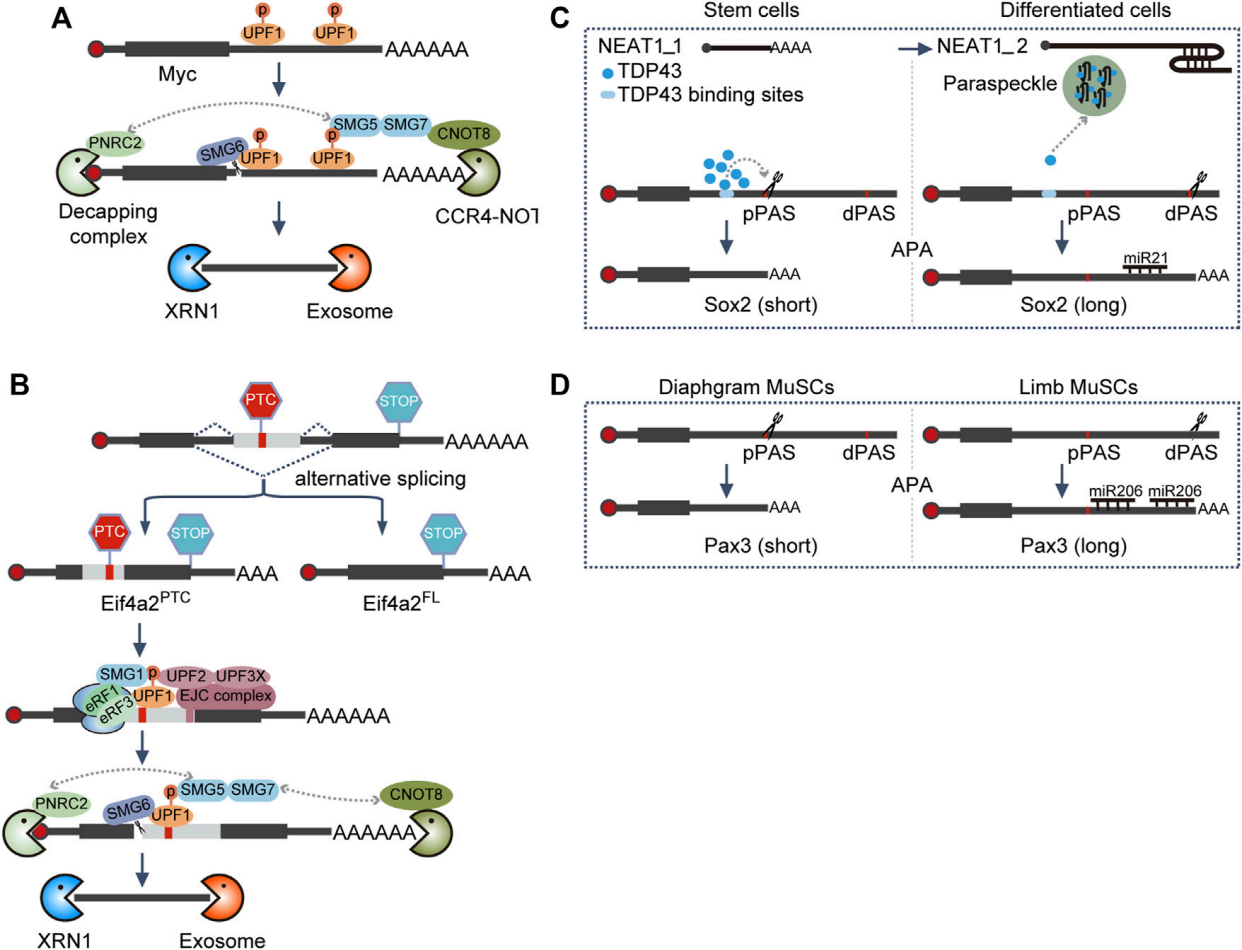
Alternative processing coupled RNA decay. **(A)** 3′ UTR-mediated NMD decay. Phosphorylation of UPF1, present on 3′ UTR, is a prerequisite for NMD activation, which in turn will recruit SMG5-SMG7 heterodimer and SMG6. NMD, non-sense-mediated RNA decay; SMG6, RNA endonuclease in the NMD pathway; CCR4-NOT, RNA deadenylation complex; PNRC2, a coactivator for RNA decapping, XRN1, 5′-to-3′ RNA exonuclease; exosome**,** 3′-to-5′ RNA exonuclease complex. **(B)** EJC-mediated NMD decay. PTC, located ≥50–55 nt upstream of an exon–exon junction, will trigger translation termination and activate the NMD pathway to phosphorylate UPF1, which in turn will recruit the SMG5-SMG7 heterodimer and SMG6. PTC, premature termination codon; STOP, normal stop codon; EJC, exon junction complex, which is assembled ∼24 nt upstream of the exon–exon junction, following splicing. **(C)** APA-mediated RNA degradation regulation of Sox2. TDP43 binds to the element upstream of the pPAS of Sox2 and enhances pPAS processing. Upon differentiation, the transition from NEAT1_1 to NEAT1_2 will facilitate the formation of paraspeckles, ultimately leading to the sequestration of TDP43 and the utilization of dPAS of Sox2. NEAT1_1 and NEAT1_2 are short and long isoforms from gene *NEAT1*, respectively. pPAS, proximal polyA site; dPAS, distal polyA site; miR, microRNA; APA, alternative polyadenylation. **(D)** APA-mediated RNA degradation regulation of Pax3. MuSCs, muscle stem cells.

Compared with the wide-type cells, *Smg6* KO mESCs displayed almost unaltered morphology and proliferation rate, suggesting SMG6 was dispensable for self-renewal maintenance. On the contrary, its differentiation potential was severely impaired, which could be recapitulated by the knockdown of other NMD factors (Li et al., 2015). Follow-up experiments revealed that NMD could target *c-Myc* for degradation through its 3′ UTR (Figure 3A). Hence, *c-Myc* is upregulated, which in turn blocks mESC differentiation upon *Smg6* KO (Cartwright et al., 2005; Li et al., 2015).

When *Smg5*, *Smg6*, and *Smg7* were knocked out independently in mESCs in another study, all these modified cell lines exhibited variable but pronounced impairment in differentiation (Huth et al., 2022), consistent with the previous study (Li et al., 2015). However, the authors did not observe an increased *c-Myc* level in NMD-deficient ESCs. Instead, they identified that *Eif4a2* was the NMD *bona fide* target responsible for differentiation delay (Huth et al., 2022). Mechanistically, *Eif4a2* could generate two isoforms through alternative splicing, one full-length isoform (Eif4a2^FL^) and the other PTC-containing isoform (Eif4a2^PTC^). NMD deficiency stabilized the Eif4a2^PTC^ transcript to produce a truncated protein Eif4a2^PTC^ (Figure 3B). Eif4a2^PTC^ protein can specifically interact with the mTORC1 negative regulator TSC2 and dampen its activity. Thus, the mTORC1 activity increases and the translation rate is elevated in NMD-deficient ESCs, resulting in a delayed differentiation phenotype (Huth et al., 2022).

#### Alternative polyadenylation coupled RNA decay in cell fate decision

More than 70% of mammalian genes undergo alternative polyadenylation (APA), which could generate transcripts encoding different proteins or with different 3′ UTR lengths (Derti et al., 2012; Hoque et al., 2013). Longer 3′ UTR isoforms usually contain additional binding sites for RBPs or miRNAs and hence tend to exhibit differential stability, translation efficiency, or cellular localization compared with their shorter counterparts (Tian and Manley, 2017). Now, we know that APA is dynamically regulated and broadly engaged in cell fate transition, partially through the control of RNA decay (Sommerkamp et al., 2021).

By coinciding with the exit of pluripotency toward differentiation for both human ESCs (hESCs) and mESCs, the *NEAT1* gene switches from the short isoform NEAT1_1 in ESCs to express the long, full-length isoform NEAT1_2 (Modic et al., 2019), a scaffold RNA necessary for paraspeckle formation (Clemson et al., 2009; Sasaki et al., 2009; Sunwoo et al., 2009; Modic et al., 2019). TDP-43 will then be sequestered into the paraspeckles by NEAT1_2, resulting in the reduction of the available TDP-43. Thus, the ability of TDP43 to enhance proximal polyA site processing is lost, and the longer 3′ UTR transcripts of SOX2 would be favored in differentiated cells, which can be further targeted by miR-21 for degradation; thereby, the pluripotency factor SOX2 is suppressed, and the dissolution of pluripotency is promoted (Figure 3C) (Modic et al., 2019).

Likewise, although miR-206 exhibits similar expression levels in both limb and diaphragm muscle stem cells (MuSCs), it only downregulates the target gene *Pax3* expression in limb MuSCs. As *Pax3* preferentially chooses the proximal polyA site (pPAS) during APA in diaphragm MuSCs to circumvent the function of miR-206, thus a high PAX3 level is sustained to support the proliferation of diaphragm MuSCs (Figure 3D) (Boutet et al., 2012).

### lncRNA decay coupled transcription regulation in cell fate decision

All the examples described previously focus on the roles of protein-coding gene decay in cell fate decisions. Given the crucial role of lncRNA in cell differentiation and development (Fatica and Bozzoni, 2014), despite being poorly characterized, the decay of lncRNA should also be involved in cell fate decisions.

**FIGURE 4:**
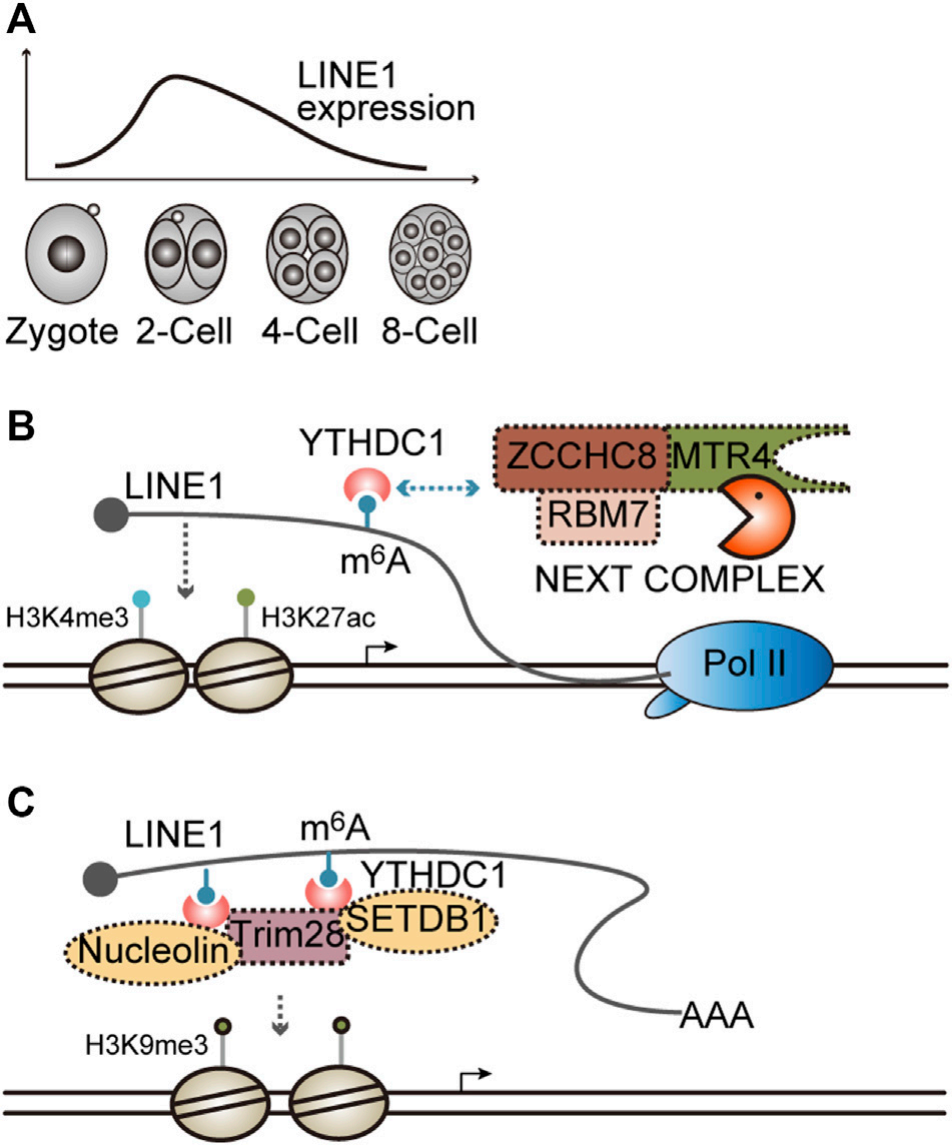
Functions and the degradation of LINE1 RNA. **(A)** Dynamic expression of LINE1 in mouse preimplantation embryos. LINE1, long interspersed nuclear element. **(B)** LINE1 RNA promotes an open chromatin state and is regulated by the NEXT complex. LINE1 can recruit histone modifiers that install activation marks H3K4me3/H3K27ac. LINE1 is modified by m^6^A modification and can be recognized by the m^6^A modification reader YTHDC1, which in turn recruits the NEXT complex to facilitate the decay of LINE1. m^6^A, N6-methyladenosine; NEXT, nuclear exosome targeting complex, is an adapter for RNA exosome. **(C)** LINE1 RNA facilitates the formation of a close chromatin state. LINE1 is modified by m^6^A modification and can be recognized by m^6^A modification reader YTHDC1, which in turn recruits Trim28/nucleolin or the Trim28/SETDB1 complex to facilitate the disposition of repressive mark H3K9me3.

The retrotransposon long interspersed nuclear element-1 (LINE1) is transcriptionally activated in mouse preimplantation development, especially at the two-cell (2C) stage (Figure 4A) (Jachowicz et al., 2017). When LINE1 expression is silenced through transcription repression immediately after fertilization, most of the embryos arrest at the 2C stage, which can be recapitulated through antisense oligo- (ASO)-mediated knockdown of LINE1 RNA, indicating the crucial role of LINE1 RNA in early embryogenesis (Jachowicz et al., 2017; Percharde et al., 2018). However, if LINE1 is enforced to express at a higher level beyond the 2C stage when LINE1 is naturally downregulated (Figure 4A), half of the embryos fail entry into the blastocyst stage (Jachowicz et al., 2017), suggesting LINE1 RNA should be maintained at a proper level to sustain mouse preimplantation embryogenesis.

Notably, LINE1 can be targeted for degradation by the NEXT complex in mouse ESCs and embryos. The depletion of Zcchc8, the scaffold subunit of the NEXT complex, will lead to LINE1 upregulation and developmental defects in mice (Wu et al., 2019). Furthermore, LINE1 is modified by m^6^A methylation, which can be recognized by the nuclear m^6^A reader YTHDC1 and then enhances the association between the NEXT complex and LINE1, thereby accelerating LINE1 degradation (Liu et al., 2020; Wei et al., 2022). When *Fto*, the m^6^A demethylase, is knocked out, the LINE1 m^6^A level is elevated, whereas its expression level is reduced accordingly. Moreover, Mice with *Fto* KO exhibit developmental defects analogous to *Zcchc8* KO (Wu et al., 2019; Wei et al., 2022). Mechanistically, it was demonstrated that LINE1 is essential for maintaining a global open chromatin state. An elevated LINE1 level results in greater chromatin accessibility, whereas a reduced LINE1 level causes chromatin condensation, which can be reflected by altered histone modifications (Figure 4B) (Jachowicz et al., 2017; Wu et al., 2019; Wei et al., 2022). Collectively, these results indicate that LINE1 is dynamically expressed during early development. Its degradation, at least partially, may contribute to the gradual chromatin compaction that occurs naturally in developmental progression, thereby ensures the ordered developmental program.

Interestingly, it was demonstrated in another two studies that the binding of YTHDC1 to LINE1 recruits Nucleolin/Trim28 or SETDB1/Trim28 complex to facilitate the deposition of H3K9me3 and then silences target gene expression, such as Dux, a master regulator of the 2C-specific transcriptome (Figure 4C) (Chen et al., 2021; Liu et al., 2021), implying that the function of LINE1 RNAs at different genomic loci may rely on the recruited effector proteins. However, how this difference is achieved remains elusive.

## Conclusion and perspectives

RNA degradation machineries, composed of RNA exonucleases, endonucleases, and other co-factors, are involved in the processing and maturation of snoRNA, snRNA, and rRNA, among others, and clearing of aberrant mRNAs with PTCs or those without stop codons (Puno et al., 2019; Wolin and Maquat, 2019). Beyond these roles in RNA processing and quality control, RNA degradation is actively implicated in the control of RNA quantity and hence the regulation of gene expression in diverse physiological activities (Tomecki and Dziembowski, 2010; Schoenberg, 2011; Akira and Maeda, 2021).

RNA degradation is especially important in cell fate decisions because rapid shifts in the mRNA and protein constitution during the transition between different cell states require activating new gene expression programs, meanwhile silencing the old ones. RNA degradation can independently clear specific pre-existing RNAs associated with the previous cell type (Akira and Maeda, 2021), or it can synergize with transcriptional repression to consolidate the silencing effect (Yamaji et al., 2017; Garland et al., 2022; Zhou et al., 2022). The coordination of RNA synthesis and RNA decay determines cell identity and plasticity. It is evidenced that the modulation of RNA decay by additional expression of certain miRNAs with transcription factors can significantly enhance the reprogramming efficiency (Judson et al., 2009; Liao et al., 2011), highlighting the important role and potential implication of RNA degradation control.

RNA deadenylation seems to be the initial and rate-limiting step in RNA degradation. RBPs (Yu et al., 2016; Yamaji et al., 2017; Sha et al., 2018; Zhao et al., 2020), small RNA (Wang Y. et al., 2008; Melton et al., 2010; Barckmann et al., 2015), RNA modification (Batista et al., 2014; Aguilo et al., 2015; Geula et al., 2015; Du et al., 2016), and NMD (Tomecki and Dziembowski, 2010; Schoenberg, 2011; Huth et al., 2022) can facilitate RNA degradation and alter the cell fate *via* the recruitment of the CCR4–NOT deadenylation complex. Although small RNA and NMD can trigger endonucleolytic cleavage of RNA (Valencia-Sanchez et al., 2006; Tomecki and Dziembowski, 2010; Schoenberg, 2011), no reported RNA endonuclease, analogous to the Regnase protein in the immunological system (Akira and Maeda, 2021), can function independently to regulate the cell fate. Maybe strategies developed to systematically map the endonucleolytic sites could enable us to identify such potential endonucleases (Karginov et al., 2010; Ibrahim and Mourelatos, 2019; Tang et al., 2022). Conversely, the RNA endonuclease complex can be co-transcriptionally loaded to cleave the nascent RNAs and trigger transcription termination (Elrod et al., 2019; Tatomer et al., 2019; Stein et al., 2022; Zhou et al., 2022); whether this or similar RNA degradation pathways are applied in regulate the cell fate awaits further investigation.

During the transition between different cellular states, the number of RBPs or miRNAs is dynamically regulated to control RNA degradation (Wang Y. et al., 2008; Melton et al., 2010; Liu et al., 2016; Yu et al., 2016; Yamaji et al., 2017; Sha et al., 2018; Zhao et al., 2020). As signal pathways are integrated to control the activity of transcription factors in pluripotency cells (Li and Belmonte, 2017), the connection between RNA degradation and signal pathways in other systems is also clear (Thapar and Denmon, 2013; Akira and Maeda, 2021). How signal pathways are linked with RNA degradation pathways to regulate cell fate is also of specific interest.
