# Supplementary material for: Control of RNA degradation in cell fate decision
Source: Front Cell Dev Biol. 2023 Mar 21;11:1164546. doi: 10.3389/fcell.2023.1164546 (PMC10070868; doi:10.3389/fcell.2023.1164546)
Supplement: Supplementary file 1 [file Table1.DOCX]

**Supplemental Table1：Major RNA degradation machineries and the co**-**factors**

Component: protein name in *Homo sapiens* and budding yeast *Saccharomyces cerevisiae*

| Complex/protein | Component (homolog in *Saccharomyces cerevisiae*) | | Catalytic activity | | Localization |
| --- | --- | --- | --- | --- | --- |
| 5'-to-3' RNA exonuclease | | | | | |
| XRN1 | | XRN1 (Xrn1) | processive exonuclease | cytoplasm | |
| XRN2 | | XRN2 (Rat1) | processive exonuclease | nucleus | |
| 3'-to-5' RNA exonuclease | | | | | |
| RNA exosome | | EXOSC1(Cls4) | - | cytoplasm and nucleus | |
|  |  | EXOSC2(Rpp4) | - | cytoplasm and nucleus | |
|  |  | EXOSC3(Rpp40) | - | cytoplasm and nucleus | |
|  |  | EXOSC4(Ski6) | - | cytoplasm and nucleus | |
|  |  | EXOSC5(Rpp46) | - | cytoplasm and nucleus | |
|  |  | EXOSC6(Mtr3) | - | cytoplasm and nucleus | |
|  |  | EXOSC7(Rpp42) | - | cytoplasm and nucleus | |
|  |  | EXOSC8(Rpp43) | - | cytoplasm and nucleus | |
|  |  | EXOSC9(Rpp45) | - | cytoplasm and nucleus | |
|  |  | EXOSC10(Rpp6) | distributive exonuclease | most in the nucleus | |
|  |  | DIS3(Dis3) | processive exonuclease  endonuclease | most in the nucleus | |
|  |  | DIS3L(Dis3) | processive exonuclease | cytoplasm | |
| RNA exosome co-factors | | | | | |
| NEXT complex | | MTR4(Mtr4) | - | nucleus | |
|  |  | RBM7 | - | nucleus | |
|  |  | ZCCHC8 | - | nucleus | |
| PAXT complex | | MTR4(Mtr4) | - | nucleus | |
|  |  | PABPN1 | - | nucleus | |
|  |  | ZFC3H1 | - | nucleus | |
| Decapping | | | | | |
| Decapping holoenzyme | | DCP2(Dcp2) | decapping activity | most in the cytoplasm | |
|  |  | DCP1(Dcp1) | - | most in the cytoplasm | |
| Decapping co-factors | | PNRC1(Edc1) | - | most in the nucleus | |
|  | | PNRC2(Edc2) | - | P body in the cytoplasm | |
|  | | EDC3(Edc3) | - | P body in the cytoplasm | |
|  | | EDC4 | - | P body in the cytoplasm | |
|  | | TTL(Pby1) | - | P body in the cytoplasm | |
|  | | PATL1(Pat1) | - | most in the cytoplasm | |
|  | | 4E-T | - | most in the cytoplasm | |
|  | | LSM1-7(Lsm1-7) complex | - | P body in the cytoplasm | |
|  | | LSM14(Scd6) | - | P body in the cytoplasm | |
|  | | DDX6(Dhh1) | - | P body in the cytoplasm | |
| Deadenylation | | | | | |
| PAN2–PAN3 complex | | PAN2(Pan2) | distributive exonuclease | cytoplasm and nucleus | |
|  |  | PAN3(Pan3) | - | cytoplasm and nucleus | |
| CCR4–NOT complex | | CNOT1(Not1) | - | cytoplasm and nucleus | |
|  |  | CNOT2(Not2) | - | cytoplasm and nucleus | |
|  |  | CNOT3(Not3/5) | - | cytoplasm and nucleus | |
|  |  | CNOT4(Not4) | - | cytoplasm and nucleus | |
|  |  | CNOT6/6L(Ccr4) | processive exonuclease | cytoplasm and nucleus | |
|  |  | CNOT7/8(Pop2) | distributive exonuclease | cytoplasm and nucleus | |
|  |  | CNOT9(Caf40) | - | cytoplasm and nucleus | |
|  |  | CNOT10 | - | cytoplasm and nucleus | |
|  |  | CNOT11 | - | cytoplasm and nucleus | |
